# Extended cancer registry data for real-world evidence in bladder cancer: an update on the Prospective Bladder Cancer Infrastructure

**DOI:** 10.1016/j.esmorw.2026.100772

**Published:** 2026-09-04

**Authors:** F.M. Uunk, J. Bosveld, J.L. Boormans, A.G. van der Heijden, L.A. Kiemeney, N. Mehra, R.P. Meijer, B.B.M. Suelmann, E.P.A. Janssen, A. Richters, K.K.H. Aben, ProBCI Study Group, Tom J.H. Arends, Saskia van der Meer, Filip J.M. Delaere, André N. Vis, Carl J. Wijburg, Ilze E.W. van Onna, Diederik M. Somford, Bart Rikhof, Bart P. Wijsman, Thomas Kerkhofs, Joan van den Bosch, Annemarie M. Leliveld-Kors, Barbara M.A. Schout, Gerd-Jan Molijn, Luc A.J. Roelofs, Martijn G. Steffens, Tom J.N. Hermans, Marleen L. Duizer, Harm H.E. van Melick, Arnold Baars, Aart Beeker, Arnoud W. Postema

**Affiliations:** 1 Department of Research and Development, Netherlands Comprehensive Cancer Organisation, Utrecht, The Netherlands; 2 Department of Medical Oncology, Radboud University Medical Center, Nijmegen, The Netherlands; 3 Department of Oncological Urology, University Medical Center Utrecht, Utrecht, The Netherlands; 4 Department of Urology, Erasmus Medical Center Cancer Institute, Rotterdam, The Netherlands; 5 Department of Urology, Radboud University Medical Center, Nijmegen, The Netherlands; 6 Department of IQ Health, Radboud University Medical Center, Nijmegen, The Netherlands; 7 Department of Medical Oncology, University Medical Center Utrecht, Utrecht, The Netherlands

**Keywords:** bladder cancer, cancer registry, cohort, nationwide, population-based, real-world

## Abstract

**Background:**

To advance clinical research and innovation in bladder cancer care, the Dutch Prospective Bladder Cancer Infrastructure (ProBCI) was established as a nationwide platform and has now been operational for 5 years.

**Materials and methods:**

ProBCI is a population-based cohort including all patients diagnosed since 2020 with high-risk non-muscle-invasive bladder cancer (HR-NMIBC), muscle-invasive bladder cancer (MIBC), or metastatic bladder cancer (mBC) in the Netherlands, comprising a Full and an Active Cohort. The full cohort includes all eligible patients, from whom core (HR-NMIBC and MIBC) or extended (mBC) clinical data and follow-up are collected within the Netherlands Cancer Registry (NCR). The active cohort is a subset of the full cohort and consists of patients who provided informed consent for additional collection of patient-reported outcomes and biomaterials alongside detailed clinical data and follow-up until death.

**Results:**

As of 1 January 2026, the ProBCI full cohort comprised 20 737 patients. A growing number of participating hospitals (now 24) have been prospectively recruiting patients in the active cohort, with 1519 patients included to date. Among these, 73% completed baseline questionnaires (optional component) and 91% provided blood samples. Data from both cohorts are used by various studies including population characterizations, biomarker validation, and as concurrent control groups for single-arm trials.

**Conclusions:**

ProBCI has evolved into a uniquely positioned research infrastructure, with data collection embedded in the NCR, providing high-quality, high-granularity data and biomaterials to address diverse research questions. ProBCI demonstrates that nationwide collaboration can generate high-quality resources that support bladder cancer research and contribute to future improvements in patient outcomes.

## Highlights

• ProBCI is a nationwide BCa cohort embedded in the NCR.
• The population-based full cohort includes longitudinal data for all patients with BCa.
• The informed consent-based active cohort also includes PROMs and biomaterials.
• Collected data support diverse research, e.g. epidemiological and biomarker studies.
• ProBCI shows the value of integrated registry-based data for BCa research.

## Introduction

Population-based cancer registries are primarily established to support cancer surveillance. Their core objectives are to systematically monitor cancer incidence, prevalence, mortality, and survival within defined populations, thereby informing public health policy, resource allocation, and evaluation of cancer control strategies.^1^ Through standardized and nationwide data collection, registries provide an essential overview of the cancer burden and its trends over time.

To fulfill this surveillance function, the Netherlands Cancer Registry (NCR) captures information on patient and tumor characteristics, disease stage, initial treatment, and vital status of all patients diagnosed with cancer in the Netherlands.^2^ To date, clinical follow-up is available for only a small subset of patients. Although the NCR data are highly valuable for population-level analyses and benchmarking across hospitals, they often lack clinical granularity and longitudinal scope. As a result, cancer registry data alone may be insufficient to address more complex or highly specific clinical research questions, for example, those related to disease recurrence/progression, patient-reported outcome measures (PROMs), biomarker studies (e.g. fibroblast growth factor receptor or circulating tumor DNA testing), treatment sequencing after reimbursement of new regimens, or effectiveness of novel therapies in routine clinical practice.

To overcome these limitations for bladder cancer, the Prospective Bladder Cancer Infrastructure (ProBCI) was initiated in 2018.^3^ ProBCI builds upon the population-based foundation of the NCR by enabling more detailed and longitudinal data collection, supporting a wide range of clinical and translational research.

In this study, we present an update on the progress of ProBCI, outlining its implementation, the data available, and summarizing the real-world evidence generated to date, illustrating the added value of this comprehensive registry-based infrastructure for bladder cancer.

## Materials and methods

### Study design and inclusion criteria

**Figure 1:**
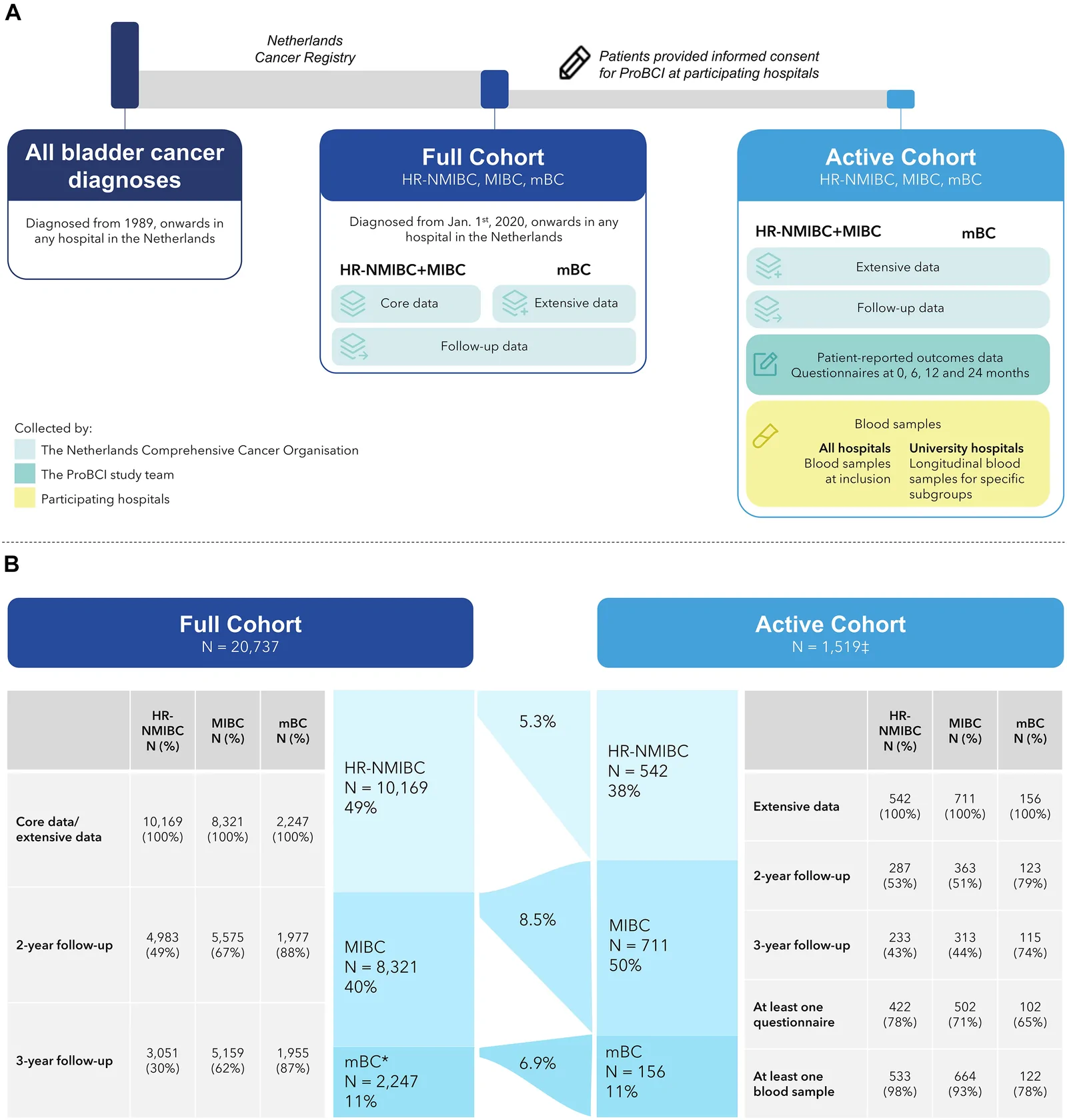
**ProBCI design.** Full cohort and active cohort (A) and number of patients included per stage group (B). ∗Additional data has been collected retrospectively for patients diagnosed with mBC between 2016 and 2019 (*n* = 1434). These patients are not included in (B). ^‡^The total number of patients in the active cohort contains recently included patients for whom tumor stage has not been documented yet (*n* = 110). Therefore, the numbers of patients in specific stage groups do not add up to the total number of patients in the active cohort. HR-NMIBC, high-risk non–muscle-invasive bladder cancer; mBC, metastatic bladder cancer; MIBC, muscle-invasive bladder cancer; ProBCI, Prospective Bladder Cancer Infrastructure.

ProBCI is an ongoing prospective cohort study in the Netherlands including newly diagnosed patients with high-risk non-muscle-invasive bladder cancer (excluding Ta tumors) (HR-NMIBC), muscle-invasive bladder cancer (MIBC), or metastatic bladder cancer (mBC). ProBCI comprises two cohorts: the full cohort and its subset, the active cohort (Figure 1A).

The full cohort consists of all patients with HR-NMIBC, MIBC, and mBC, diagnosed in any hospital in the Netherlands from 2020 onward. In addition, patients diagnosed with mBC in 2016-2019 were retrospectively included through a one-time data collection across all Dutch hospitals. For this full cohort, additional clinical variables beyond those routinely captured in the NCR are collected.

The active cohort includes patients who have been prospectively recruited since 2020 by a growing number of participating hospitals and provided broad informed consent. Currently, 6 of the 7 university hospitals, 14 of the 27 nonuniversity hospitals, and 4 of the 34 general hospitals are participating, totaling 24 participating hospitals. Although clinical data collection itself does not rely on informed consent, this consent enables linkage with other registries, extended use and enrichment of clinical data, as well as the collection and use of biomaterials, PROMs, and potential participation in other studies including trials within cohorts (TwiCs).^4^

The active cohort of ProBCI has been approved by the ethics committee NedMec (ref. no. NL70207.041.19).

### Clinical data collection

The clinical data collection of ProBCI is embedded within the framework of the NCR. The NCR is maintained by the Netherlands Comprehensive Cancer Organisation and contains data of all patients diagnosed with cancer in the Netherlands since 1989. The NCR receives automated notifications of new cancer diagnoses through the national pathology database (Palga) and the national hospital discharge database (LBZ), providing nationwide coverage of cancer diagnoses in the Netherlands. Several months after diagnosis, trained registration personnel with access to electronic health record systems in all Dutch hospitals manually extract data on patient demographics, disease characteristics (such as date of diagnosis, topography, morphology, lateralization, and stage), and primary treatment. Data are coded according to international classification systems [e.g. TNM (tumor-node-metastasis) classification,^5^ and International Classification of Diseases for Oncology^6^] and subsequently stored in the NCR data warehouse. Patients who receive care across multiple hospitals are followed longitudinally across institutions, ensuring comprehensive capture of treatment trajectories. Validation procedures are routinely carried out to check for data completeness and detect unusual or inconsistent data entries. However, items that are not consistently reported in electronic health records, such as performance status, will also be incomplete in the NCR as a result. Annual linkage with the Dutch Municipal Personal Records Database enables automated updates of vital status. Recent developments aimed at improving the efficiency of data collection have enabled the automated extraction of specific data elements (e.g. surgical interventions, laboratory values, and discharge dates, with ongoing expansion). To ensure data quality, during the implementation phase, the extracted data are manually verified.

Within the full cohort, core data and follow-up information are collected for all patients with HR-NMIBC and MIBC. These core data extend beyond standard NCR data and include additional information at diagnosis (e.g. body height and weight, performance status, and renal function) and basic follow-up on disease events (recurrence and progression) and subsequent treatments. For patients with mBC, an exception applies: although part of the full cohort, a more extensive clinical dataset is collected, including detailed longitudinal data across the disease trajectory, such as laboratory values, radiological response, and systemic treatment characteristics.

For the subset of patients with informed consent, comprising the active cohort, extensive data and comprehensive longitudinal follow-up are collected from diagnosis until death. These data are more granular than those collected in the core dataset of the full cohort. A detailed overview is provided in Table 1.

**Table 1:** Core and extensive item set

Category and data item	Core data (HR-NMIBC and MIBC patients in full cohort)	Extensive data (all patients in active cohort and mBC patients in full cohort)
Patient data		
Age, sex, socioeconomic status	x	x
Comorbidities	x	x
Vital status	x	x
Date and cause of death and source	x	x
Date of recurrences/progressions	x	x
Body height/weight	x	x
Other oncologic disease	x	x
Disease characteristics		
Date of diagnosis	x	x
Basis for diagnosis	x	x
Location/sublocation	x	x
Histology	x	x
Grade	x	x
*In situ* component	x	x
cTNM	x	x
pTNM	x	x
Number of lymph nodes harvested/positive	x	x
Location of all metastases	x	x
Basis for diagnosis all metastases	x	x
Investigations before treatment		
Performance status and ASA score	x	x
Laboratory values on hemoglobin	x	x
Laboratory values on renal function	x	x
Laboratory values on liver function		x
Laboratory values on differential WBC		x
PD-L1 expression		x
Multidisciplinary meeting		
Treatment plan		x
Reasons for deviating from treatment plan		x
Investigations after treatment		
Cystoscopy (positive/negative)		x
Radiological response		x
Hospital readmissions		x
Complications after surgical treatment	x	x
Complications after radiotherapy		x
Treatments		
Starting date of each treatment	x	x
Stop date/discharge date of each treatment	x	x
Hospital of each treatment	x	x
Transurethral resection	x	x
Bladder instillations	x	x
	including specific agent	including specific agent
Cystectomy, including urinary diversion, resection margins, and surgical approach	x	x
Lymph node dissection	x	x
External beam radiotherapy	x	x
	including setting (chemoradiation/solitary)	including setting (chemoradiation/solitary) and dosing/fractions
Brachytherapy	x	x
	including setting (chemoradiation/solitary)	including setting (chemoradiation/solitary) and dosing/fractions
Systemic therapy (neoadjuvant, adjuvant, perioperative, and palliative)	x	x
	including specific agent/regimen	including specific agent/regimen, number of cycles, adjustments, reasons for adjustments
Resection of metastasis	x	x
Radiotherapy on metastasis	x	x

ASA, American Society of Anesthesiologists; cTNM, clinical tumor, node, metastasis; HR-NMIBC, high-risk non–muscle-invasive bladder cancer; mBC, metastatic bladder cancer; MIBC, muscle-invasive bladder cancer; PD-L1, programmed death-ligand 1; pTNM, pathological tumor, node, metastasis; WBC, white blood cell count.

### Patient-reported data

Questionnaires, covering demographics, comorbidities as well as the EuroQoL 5-Dimension 5-Level,^7^^,^^8^ the European Organisation for Research and Treatment of Cancer Quality of Life Questionnaire-Core 30,^9^ the Quality of Life Questionnaire-Non-Muscle Invasive Bladder Cancer Module,^10^ and the Quality of Life Questionnaire-Muscle Invasive Bladder Cancer Module,^11^ are sent to patients in the active cohort shortly after inclusion and again at 6, 12, and 24 months after diagnosis. Delivery is via postal mail or electronic mail depending on patient preference. PROMs are centrally collected within the Patient-Reported Outcomes Following Initial treatment and Long-term Evaluation of Survivorship registry.^12^ In hospitals that manage their own PROMs collection, data are captured locally and subsequently harmonized with the centrally collected PROMs.

### Biomaterials

Blood samples for cell-free DNA and genomic DNA analyses are collected at baseline from all patients in the active cohort and centrally stored in a biobank. For patients treated at university hospitals, additional samples are collected at multiple time points to enable pre- and post-treatment analyses.^3^ For specific research questions, blood sample collection can be temporarily expanded (e.g. other time points or sample types) within targeted disease stages and/or treatment subgroups.

Patients in the active cohort also provide consent for use of residual tissue (e.g. from transurethral resections of the tumor, radical cystectomies, or biopsies) for research. These tissues samples are stored in local pathology laboratories, with reports registered in Palga according to regular standard of care. Availability is therefore not centrally tracked but tissue can be retrieved for selected subsets of the active cohort.

## Results

### Data and material collection

As of 1 January 2026, the ProBCI full cohort comprises 20 737 patients (Figure 1B). Clinical follow-up of at least 2 years after diagnosis is available for 49%, 67%, and 88% of patients with HR-NMIBC, MIBC, and mBC, respectively, while at least 3 years of follow-up is available for 30%, 62%, and 87%.

At that date, the ProBCI active cohort included 1519 patients (estimated response rate >90%), of which 551 patients were included by a university hospital. Baseline data collection has been completed for 1409 patients. Among these patients, 2-year clinical follow-up is available for 53%, 51%, and 79% of those with HR-NMIBC, MIBC, and mBC and at least 3 years for 43%, 44%, and 74%, respectively. Clinical follow-up is ongoing and will continue to mature over time.

The response rates for the quality of life (QoL) questionnaires at inclusion and at 6, 12, and 24 months were 73%, 71%, 65%, and 57%, respectively. Blood samples were collected for 94% of patients. Among patients treated in a university hospital, 20% had at least one follow-up blood sample collected. Baseline characteristics of the full and active cohorts are presented in Table 2.

**Table 2:** Baseline characteristics of the full cohort and active cohort

	Full cohort		Active cohort^a^	
	*N*	%	*N*	%
**All patients**	20 737	100	1409	100
**Sex**				
Male	15 745	76	1106	78
Female	4992	24	303	22
**Age, years**				
0-60	2413	12	234	17
61-70	5037	24	457	32
71-80	8304	40	586	42
>80	4983	24	132	9
Median (IQR)	74	67-80	71	64-76
**Charlson Comorbidity Index**				
0	8125	39	634	45
1	4760	23	325	23
>2	7285	35	441	31
Missing	567	3	9	1
**ECOG performance status**				
0	5415	26	549	39
1	2305	11	211	15
2-4	1621	8	58	4
Missing	11 396	55	591	42
**Tumor histology**				
UC	19 212	93	1355	96
Non-UC	1525	7	54	4
**HR-NMIBC subgroup**	10 169	49	542	38
**Stage**				
Tis, N0, M0	2158	21	112	21
T1, N0, M0	5911	58	307	57
T1+is, N0, M0	2100	21	123	23
**Primary treatment**				
TUR + instillation(s)	8380	82	393	73
TUR only	1133	11	13	2
RC	576	6	134	25
Other, e.g. coagulation	80	1	2	0
**MIBC subgroup**	8321	40	711	50
**Stage**				
T2, N0, M0	4594	55	411	58
T3/4, N0, M0	2196	26	176	25
Tany, N1-3, M0	1531	18	124	17
**Primary treatment**				
RC + preoperative systemic treatment	1200	14	190	27
RC	2007	24	247	35
TMT	1307	16	128	18
Radiotherapy	1248	15	51	7
Systemic therapy	461	6	52	7
Other/best supportive care only	2098	25	43	6
**mBC subgroup**	2247	11	156	11
**Stage**				
Tany, Nany, M1a	602	27	46	29
Tany, Nany, M1b	1645	73	110	71
**Primary treatment**				
Systemic therapy	829	37	113	72
Local treatment (RC/TMT/EBRT)	152	7	19	12
Other/best supportive care only	1266	56	24	15

EBRT, external beam radiotherapy; ECOG, Eastern Cooperative Oncology Group; HR-NMIBC, high-risk non–muscle-invasive bladder cancer; IQR, interquartile range; mBC, metastatic bladder cancer; MIBC, muscle-invasive bladder cancer; RC, radical cystectomy; TMT, trimodality treatment; TUR, transurethral resection of the tumor; UC, urothelial cancer.

a Patients included in the study before 1 January 2026 and who have complete baseline data.

### Ongoing research and research possibilities

ProBCI data (clinical data, PROMs, and biomaterials) support a wide range of observational and experimental research, including real-world effectiveness analyses and TwiCs. Data can be requested through the standard portal for NCR data requests (www.iknl.nl/en/ncr/apply-for-data), where all requests are reviewed by the NCR privacy board. Requests for ProBCI data are additionally reviewed by members of the ProBCI steering committee. ProBCI data have been extensively used over the past 5 years; examples of completed and ongoing studies are presented in Table 3.

**Table 3:** Research examples conducted within the ProBCI

	Examples of ongoing and completed ProBCI-based research
**Published studies**	
Detailed characterization of unselected patient populations	• Treatment and survival outcomes of patients with synchronous and metachronous mBC in the Netherlands.^13^ • Characterization, management, and outcomes of a contemporary cohort of MIBC patients (FM Uunk, unpublished data).
Comparative effectiveness	• Overall survival of patients receiving cisplatin or carboplatin for primary metastatic urothelial carcinoma of the bladder.^14^ • Real-world comparison of avelumab maintenance versus pembrolizumab at progression after first-line platinum-based chemotherapy in metastatic UC.^15^
Trials compared with clinical practice	• Comparison of patients with metastatic urothelial bladder cancer treated with pembrolizumab after platinum-based chemotherapy in a Dutch nationwide cohort and the KEYNOTE-045 trial.^16^
Guidelines versus clinical practice	• Comparison of guidelines and real-world data in mBC patients receiving first-line chemotherapy.^17^
**Ongoing studies**	
PROMs	• Evaluation of effect of high-risk recurrences on health-related QoL among patients with HR-NMIBC (study ongoing).
Concurrent control group for single-arm studies	• Two ongoing single-arm intervention studies evaluating new treatment strategies for MIBC or mBC, for which a concurrent (matched) control group is derived from the full cohort or active cohort (unpublished data).
Biomarker validation	• A biomarker study validating a tissue-based biomarker for prediction of recurrences in HR-NMIBC using residual tissue samples combined with extensive clinical data from patients in the active cohort (study ongoing). • A biomarker study evaluating a blood-based biomarker for prediction of response to immunotherapy in mBC, for which specific blood samples are collected prospectively and combined with extensive clinical data from patients in the active cohort (study ongoing).
**Additional research opportunities**	
TwiCs studies	• In a TwiCs study, patients are selected from the active cohort based on the TwiCs selection criteria and randomly assigned to an experimental arm or control arm based on the broad consent for the cohort. The control arm must always be standard of care, and therefore, no additional informed consent from the patients in the control arm has to be obtained. Patients were randomly assigned to the experimental arm must provide additional informed consent for the TwiCs. This design optimizes the benefits of randomized studies, while also improving patient participation/retention and is especially beneficial if the experimental approach is desired by patients (e.g. less invasive diagnostic procedures). An example in the Netherlands is the MEDOCC-CREATE study.^18^
Patient identification for studies from the active cohort	• Based on the broad informed consent for the active cohort, patients can be contacted to participate in other studies. By selecting patients with specific criteria (e.g. specific stages, specific treatments) from the active cohort, recruitment for other studies may be highly efficient. Examples include identifying patients who will receive adjuvant immunotherapy for sequential cfDNA testing or identifying patients who underwent new systemic regimens to obtain additional PROMs.
QoL evaluations	• Longitudinal QoL data can be used for comparative research questions regarding the differences in QoL outcomes between treatments (e.g. adjuvant treatment compared with no adjuvant treatment), research questions regarding the longitudinal profile of relevant outcomes (e.g. urinary functioning after bladder-sparing treatment), and to provide estimates to be used as inputs for health technology assessment modeling.

cfDNA, cell-free DNA; HR-NMIBC, high-risk non–muscle-invasive bladder cancer; mBC, metastatic bladder cancer; MIBC, muscle-invasive bladder cancer; ProBCI, Prospective Bladder Cancer Infrastructure; PROMs, patient-reported outcome measures; QoL, quality of life; TwiC, trial within cohorts; UC, urothelial cancer.

To illustrate the potential of ProBCI, we highlight two examples of epidemiological studies. Firstly, a comparative effectiveness study evaluated the effect of introducing maintenance avelumab after chemotherapy as standard of care, replacing pembrolizumab at disease progression, on overall survival in mBC patients, since no randomized comparison supported this guideline change. No survival improvement was observed in the era with avelumab as standard of care, suggesting that deferring treatment until progression may be equally effective while reducing treatment burden.^15^

Secondly, a nationwide, unselected cohort of patients with mBC treated with second-line pembrolizumab was compared with the KEYNOTE-045 trial population. Real-world patients, including those meeting trial eligibility criteria, had less favorable characteristics and shorter survival, underscoring discrepancies between trial populations and routine, which may affect the outcomes of treatment.^16^^,^^19^

Beyond these examples, ongoing biomarker studies within ProBCI aim to support treatment selection across disease stages and the cohorts serve as concurrent control groups for multiple single-arm studies (Table 3).

Together, these examples illustrate only part of the infrastructure’s potential. Future research may expand into areas such as (comparative) QoL outcomes, (randomized) evaluation of diagnostic procedures with or without the TwiCs study design, identification of novel therapeutic targets, and development of organoid models to support more personalized treatment strategies. International data linkage and cross-country comparisons of care are an additional area of future potential.

## Discussion

Since its initiation, ProBCI has evolved into a robust nationwide infrastructure providing comprehensive clinical data, PROMs, and biomaterials for research from a continuously expanding cohort of patients with bladder cancer. It has proven to be an efficient platform for a wide range of research initiatives enabling nationwide collaboration to improve bladder cancer outcomes.

Established at a critical time point in a rapidly changing therapeutic landscape, ProBCI provides insights into real-world practice during a period marked by the introduction of multiple novel systemic regimens for both MIBC and mBC. In this context, ProBCI is uniquely positioned to efficiently evaluate real-world effectiveness, assess outcomes in underrepresented patient populations, including older and comorbid patients, and contribute to refinement of treatments by supporting biomarker studies.

Although ProBCI has already supported multiple studies, its full potential is larger. As follow-up matures, with the first patients now reaching 5 years of follow-up, and the cohort continues to expand, new research opportunities are emerging. Increasing numbers of patients have completed questionnaires at consecutive time points, enhancing the longitudinal depth of the PROMs data and enabling robust QoL research as well as health technology assessment modeling. Although response rates remain similar or high compared with other population-based bladder cancer cohorts,^20^, ^21^, ^22^ researchers using PROMs data should consider mechanisms of missing data, which may be facilitated by availability of clinical data of patients with missing PROMs data. Additional opportunities include integration of the TwiCs design to improve efficiency of pragmatic interventional research. However, no TwiCs studies using ProBCI data have been initiated to date, whereas a sufficiently large active cohort was established. Now that the cohort has matured, the infrastructure is increasingly suitable for this approach. Although the TwiCs design offers clear advantages, it is not suitable for all research questions and careful consideration is needed to determine whether this approach is appropriate for a specific intervention. Furthermore, limited familiarity with the methodology may result in overlooked opportunities for its application, highlighting the importance of actively promoting its potential. The availability of biomaterials enables increasingly granular molecular profiling, including DNA and RNA sequencing through both standard-of-care diagnostics and within translational research. The integration of longer clinical follow-up, repeated PROMs assessments, and biomaterials provides opportunities to link molecular data with clinical outcomes, treatment sequences, PROMs, and *ex vivo* organoid drug sensitivity. This will enhance the understanding of molecularly defined disease trajectories, prognostic subgroups, and mechanisms underlying treatment response and resistance. In this way, ProBCI is evolving beyond a real-world outcomes research infrastructure into a platform for translational precision oncology research in bladder cancer as well.

ProBCI integrates comprehensive data collection within a well-established nationwide cancer registry, ensuring high data quality and completeness. Moreover, it represents a collaborative effort between researchers and clinicians, fostering knowledge exchange and the generation of new research ideas. Maintaining and further developing this infrastructure requires sustained commitment from all stakeholders, including participating hospitals, clinicians, and researchers. Continued engagement is essential to ensure nationwide coverage and data completeness, as well as to improve the efficiency of data collection, for example, through automated data extraction where feasible. Long-term sustainability also depends on stable and structurally embedded funding. To date, ProBCI has been largely supported by contributions from several pharmaceutical companies. These funding parties play no role in the assessment of data requests and the concept, execution, or reporting of the research results of studies using ProBCI data. As the importance and use of data infrastructures grow, transitioning toward more sustainable, structurally embedded funding is essential to ensure continuity and independence.

### Conclusion

Five years of operation have established ProBCI as a nationwide infrastructure integrating clinical data, patient-reported outcomes, and biomaterials for bladder cancer research. The successful development of ProBCI has been facilitated by the existing infrastructure of the NCR, which provided a strong foundation for nationwide collection and longitudinal follow-up. The cohort has supported a growing portfolio of observational and translational studies and provides a foundation for future observational and interventional research. Although opportunities remain to further optimize participation, evaluate representativeness, and increase awareness of innovative applications such as the TwiCs design, ongoing initiatives continue to strengthen and expand the infrastructure. ProBCI illustrates the value of nationwide collaboration in creating a resource that supports bladder cancer research and we welcome new collaborations to further maximize its scientific and societal impact.
